# Editor's choice

**DOI:** 10.4314/ahs.v26i1.1

**Published:** 2026-03

**Authors:** James K Tumwine

We welcome you to this March 2026 issue of African Health Sciences, in which we have selected for you 15 interesting papers.

The infectious disease section includes two COVID-19 papers that report on gastrointestinal microbial profiles of COVID-19 patients^1^; and difficulty with health care access because of fear of COVID-19 infections among women of reproductive age.^2^ These are followed by papers on tuberculosis,^3^ snake bite,^4^ monkey pox^5^, and hand washing.^6^

The next theme is mental health. Here we have a paper on pre-operative anxiety;^7^ alcohol use among displaced populations;^8^ and prevalence of dementia in Northern Uganda.^9^

The mental health treatise ends with a paper on sleep quality among hospitalised older patients in Iran.^10^

Next we have oncology papers which include colorectal carcinoma in Algeria^11^, and histopathological thyroid lesions in Nigeria^12^.

Metabolic syndrome and arterial stiffness in people of African ancestry^13^; proton pump inhibitors and risk of fractures in type 2 diabetes mellitus in menopause^14^, and medication errors in Tanzania ends this section on communicable diseases^15^.

We end the issue with an obituary on Prof Kusum Nathoo, who was a pillar of teaching and practice of Paediatrics and Child health in Zimbabwe^16^.
